# Swipes vs. Strides: How Mobile Media Use Shapes Children’s Gross and Fine Motor Skills

**DOI:** 10.3390/children12101337

**Published:** 2025-10-05

**Authors:** Michael Langlais, Valeria Veras, Faith Davidson, Ashleigh Rhyner

**Affiliations:** 1 Department of Human Sciences and Design, Baylor University, Waco, TX 76798, USA; faith_davidson1@baylor.edu (F.D.); ashleigh_rhyner1@baylor.edu (A.R.); 2 City Church, Tallahassee, FL 32312, USA; valeriaveras456@gmail.com

**Keywords:** mobile media use, children, developmental milestones, gross motor skills, fine motor skills, parenting, co-viewing

## Abstract

Many policy recommendations state that children aged 2 to 5 should not spend more than an hour per day in front of a screen; however, these recommendations are challenged as technology use becomes more mobile and accessible to young children. **Background/Objectives:** The goal of this study is to examine the relationship between children’s and parents’ mobile media use (i.e., smartphones and tablets) and children’s developmental milestones, including their fine motor, gross motor, and personal social skills. **Methods:** Data for this study comes from two child development centers in the southwestern United States, one serving predominantly middle class families and another serving low-income families (N = 63). Parents completed online surveys regarding their own and their children’s social media use by uploading screenshots of their own and their children’s mobile media device (if applicable) over the last 24 h. Parents identified how many minutes they spent using social media, how many minutes their child spent using social media, and how many minutes their child spent watching television. To capture children’s developmental milestones, parents completed the ages and stages questionnaire (ASQ3), which measures children’s fine motor skills, gross motor skills, and social skills. **Results:** Correlation and regression analyses revealed that parent mobile media use was positively associated with children’s gross motor and personal social skills (*B* = 0.38 and 0.32, respectively, *p* < 0.05; *R*^2^ = 0.09–0.19) and children’s television viewing was negatively associated with children’s gross motor skills (*B* = −0.30, *p* < 0.05). **Conclusions:** Children’s mobile media may have different consequences for children’s developmental milestones compared to television, and parents’ mobile media use may be more associated with children’s developmental milestones than children’s own use of these devices.

## 1. Introduction

The rate of exposure of infants to smartphones has evolved from 25% to approximately 75% [1,2], and rates are higher for young children 2 to 5 years old, who spend an average of two hours per day on smartphones [3]. In 2016, the American Academy of Pediatrics announced recommendations stating that digital media use in children younger than 24 months should be avoided, except video-chatting, because interacting with others via video can promote language and cognitive development [4]. However, the research is inconsistent with these recommendations. Studies have shown that children can benefit from screen time if it incorporates conversation with adults and there are opportunities for learning [5,6]. Other studies, though, show that when children are with parents who continuously use mobile technology, it takes away from active family relationships [7]. In contrast ot these findings, Downey and Gibbs found no difference in children’s skills regardless of screen time [8]. Given these inconsistencies, it is clear that research on mobile media and children’s development has not caught up with its rate of adoption.

The goal of this study is to investigate the relationship between parents’ and children’s use of mobile technology (such as smartphones and tablets) for children’s (ages 0 to 5) developmental milestones—specifically their fine motor, gross motor, personal social, problem solving, and communication skills. The sample consisted of parents with at least one child between the ages of 0 and 5 years, spanning two key ontogenetic periods: infancy/toddlerhood (0–2 years), characterized by rapid sensorimotor, language, and attachment development, and early childhood/preschool age (3–5 years), marked by increasing autonomy, socioemotional growth, and emerging school readiness. This study is novel in that it simultaneously examines both parents’ and children’s mobile technology use in relation to multiple domains of developmental milestones in early childhood, encompassing the distinct ontogenetic periods of infancy, toddlerhood, and early childhood. This research is important because understanding how mobile media use by parents and children influences developmental outcomes can inform data-based guidelines to support child development, shape parenting practices, and support policies that promote healthy child development.

## 2. Literature Review

Over recent years, there has been a noticeable surge in the use of media and electronic devices within households [9]. This shift has sparked growing concern and curiosity about the effects of screen time on young children, particularly those aged 0 to 5. At this critical stage of development, children require active engagement and attention to foster fine motor skills, language acquisition, and social–emotional growth [10]. Yet, many children under the age of three are given their own personal tablets despite the recommendations from the American Academy of Pediatrics discouraging media use by children younger than 2 in totality and recommending limiting children 2 to 5 to no more than one hour of screen time a day [9]. These recommendations are based on studies that focus exclusively on time spent on a device, ignoring how the device is used and who the device is used with, if not alone. Recent research has highlighted the potential benefits of co-view, where parents and children engage with media together [11]. Therefore, the role of mobile technology for children’s development should consider elements beyond the amount of time spent on a smartphone or tablet.

This study is situated in the interactional theory of childhood problematic media use [12]. This theory defines problematic media use as behavior that impairs current or future functioning, distinguishing it from normal variation by identifying it as a pathological level of behavior, by focusing on media use, whether the use is maintainable or problematic, the damages possible, and how to intervene, if necessary. This theory came out of the realization that the growth of mobile devices and the decreasing age at which children receive their first device has potential to risk the health of a child’s development [12]. Through the lens of this theory, children’s extensive time spent on a mobile device can become problematic to the point that it disrupts children’s physical and social development. Additionally, this study is aligned with the main ideas of Vygotsky’s sociocultural theory of development, which highlights the role of social interaction and culture in learning [13,14]. A key concept in this theory is the “zone of proximal development,” which refers to the range of tasks that learners can perform with guidance but not yet independently, highlighting the role of a more knowledgeable individual in supporting skill acquisition beyond the learner’s current capabilities. The support provided, referred to as scaffolding, allows children to master skills that are just beyond their realm of ability. Using this theory, co-using digital media with a caregiver can scaffold mobile media to help support children’s development. This logic assumes that if parents are involved with children’s viewing, that children will gain many benefits from co-viewing mobile media content.

### 2.1. Early Childhood Developmental Milestones

The development of motor skills in children predicts future physical and physiological well-being [15]. Although mobile media continues to be integrated into the lives of children, screen media use may have adverse effects on children’s health in the long term, such as increased risk of obesity, sleep irregularities, and poor academic performance [10]. Physical activity and gross motor skills share a reciprocal relationship; gross motor skills are essential for engaging in physical activity, and when physical activity is lacking, gross motor development also declines as well as the confidence and foundational skills necessary for maintaining an active lifestyle later in life [16]. Additionally, the development and mastery of body movement greatly benefits from direct help from the caregiver [17]. Caregivers can provide the knowledge and support needed for children to master the appropriate gross motor skills for their stage in development.

Gross motor skills, representing movements of the limbs and torso, are negatively associated with high screen media, whereas fine motor skills are less impacted [18]. For instance, there is a negative relationship between hours of television watched when children are 29 months old and gross motor movement at age 5 [19,20]. Alternatively, fine motor skills, which pertain to the control and coordination of the distal musculature of the hands and fingers [21], are improved by manipulating objects in the environment (e.g., grasping, touching, in-hand manipulation) [19]. Although television use may not promote fine motor skill development, interactive tools like mobile devices may provide benefits in terms of developing fine motor movements [22].

Additionally, children’s environmental experiences, which includes the amount and quality of care, stimulation and interaction they receive, will influence children’s cognitive and neural development [23]. A cognitively stimulating environment engages different senses and includes social interactions that can support children’s development. While cognitive stimulation may involve independent exploration, for infants and young children, it often involves engagement with an adult who can provide learning opportunities and guide attention [23]. There is some support that mobile technology disrupts opportunities for children to interact with their environment, and therefore, may not benefit cognitive development [6].

Evidence suggests that there is a relationship between excessive early screen exposure during critical stages of development and development delays [24]. Research shows that excessive screen exposure may lead to deficits in a child’s attention, communication skills, and social behavior [10]. Screen time also replaces the activities known to support children’s brain development: bonding, movement, eye contact, face-to-face verbal interactions, loving touch, exercise, free play, and exposure to nature and the outdoors [9]. Reduced exposure to these factors will discourage children from these activities and direct them to others that are more intellectually and physically passive, which have the potential to negatively influence cognition.

In addition, there is a fear that interactive media may displace sensorimotor activities that support development of visual, motor, and social skills important to later success [3,6]. This displacement may reduce opportunities for children to engage in hands-on play, outdoor exploration, and face-to-face social interactions—all of which are critical for developing coordination, problem solving, and emotional regulation [3,12]. Over time, limited engagement in these real-world experiences could hinder foundational developmental processes that digital media alone cannot adequately replace.

### 2.2. Parent Use of Mobile Media Use and Children’s Development

Data from the Pew Research Center shows that 68% of parents feel that their use of mobile devices sometimes or often gets in the way of their parenting skills, which is relatively consistent based on age, ethnicity, and education [25]. Although parents voice concerns about their child’s mobile media use, parents often engage in excessive mobile media use at home [26,27]. In a qualitative investigation of parents’ attitudes of allowing their children to use mobile media, one parent said, “I am torn because I know other parents are letting their children [use] tablets and phones at very young ages, and I know that they will suddenly be unleashed when they go to school because the school gives out tablets to every incoming student, so am I hurting them or putting them at a disadvantage by [not giving them a tablet]?” [28]. This parent’s concern is validated, given that research typically recommends against children’s exposure to technology, and yet children are supposed to have some digital literacy to be successful in school and in the future.

Nevertheless, parent’s own use of mobile media, rather than their attitudes concerning their children’s use of mobile media, may be a strong predictor of their children’s use [27,28,29]. McDaniel and Radesky found that a mother and father’s excessive mobile media use predicted technoference (interference of technology within a relationship) between them and their children, and that maternal technoference predicted increases in children’s problem behaviors [30]. Lederer et al. found that mothers’ smartphone use predicted fewer statements and responses by them to their children, and that mothers missed children’s bids for assistance more frequently. In an observation of parent–child dyads at parks, 59% of parents used their smartphone at the park, and 35% of these parents engaged with their smartphone every one to five minutes [31]. Elias et al. found that parents ignore their children’s interactions to a degree that their children’s safety and emotional needs may be at risk [32]. Plowman and Stephen identified four critical factors within the home environment that shape a child’s experience with technology: (1) the family’s overall attitude toward using technology as an educational tool, (2) parental perspectives on supporting their child’s learning and development, (3) the presence of siblings and their influence, and (4) the time constraints and unique circumstances experienced by parents, including their own digital habits and stress levels [33]. These factors create the ecosystem through which a child engages with media. Essentially, parents’ media habits serve as direct models for children, influencing their attitudes toward and interactions with technology [3,27]. Overall, if parent smartphone use interrupts opportunities for parent–child interaction, parental responsiveness is likely to decline.

### 2.3. Parent–Child Co-View of Media

Although parents’ use of mobile media could hinder child development, recent studies have shown that parents watching and engaging in mobile media devices with their child can help promote the child’s development [34,35,36]. For example, Kiraly et al. encouraged parents to engage in media consumption with children during the pandemic given that many families resorted to consuming technology during that time [35]. Watching content with children is particularly useful when caregivers encourage their children to ask questions and help children understand what they are seeing [9,37].

Parental involvement in children’s media consumption has been shown to significantly influence their learning, play, and social development [38]. Research indicates that co-viewing can enhance children’s ability to respond accurately to content and support developmentally appropriate learning [9]. By 24 months, children can acquire new vocabulary through interactive touchscreens and video chats, while by 25 months, they are capable of learning novel words from touchscreen interactions [37]. Furthermore, studies suggest that media without parental involvement, regardless of educational content, is often ineffective for learning [38,39]. For example, passive exposure to educational DVDs over a month failed to facilitate new word learning in toddlers [39]. In contrast, the highest levels of learning occurred in conditions where parents actively taught the same words during everyday interactions, without video support. These findings underscore the conclusion that screens, when used in isolation, may not reliably support early learning.

On the other hand, parents and children using media together could replace meaningful face-to-face interactions that support child development. According to the displacement hypothesis [34], time spent with technology or media may displace and decrease meaningful parent–child connections. A primary area of concern regarding parental screen distractions is what effect these displaced parent–child interactions could have on children’s development [40]. Enhanced technology use can distract parents from parent–child interactions, which may lead to less responsive and sensitive parents [41,42]. According to this logic, co-viewing mobile technology may not be beneficial for the development of children’s motor and social skills.

### 2.4. The Present Study

The existing literature highlights significant interest in the impact of screen media on early childhood development, particularly regarding gross motor and fine motor skill development. Studies have explored the implications of increased screen time, the role of parent–child co-viewing and how it can help parents use media to further develop their young children or interfere with meaningful parent–child interactions [6,18,40]. Essentially, many ideas have stemmed from research on television with less research on mobile media. There is also comparatively less research analyzing direct relationships between screen and media exposure and motor development in children aged 0–5. Theoretical and empirical evidence suggests a negative association between media exposure and the development of gross motor skills, interpersonal/social abilities, and communication milestones. Conversely, media use may be supportive if co-viewing with a parent occurs. Given this information, we propose the following hypotheses:

**Hypothesis** **1:** *Children’s mobile media use will be negatively associated with their gross motor and personal–social developmental milestones and positively associated with their fine motor skills.*

**Hypothesis** **2:** *Parent’s mobile media use will be negatively associated with children’s gross motor and personal–social use, and positively associated with their children’s fine motor skills.*

**Hypothesis** **3:** *Parent–child co-viewing of mobile media will be positively associated with children’s personal–social and fine motor developmental milestones, and negatively associated with their gross motor skills.*

## 3. Methods

### 3.1. Procedures

Participants in this study were required to be parents of at least one child between the ages of 0 and 5. The families for this study were recruited from two childcare facilities in the southern central United States. The first facility was a small university-based childcare facility predominantly serving middle-income families (n = 22) and the second facility was a large faith-based facility serving predominantly low-income families (n = 41). Directors of these childcare facilities shared the emails of parents with the principal investigator, who emailed them information about the study. These emails stated the goal of the study, inclusion criteria (parents had to be at least 18 years old, and their child had to be between 0 and 5), what was involved with the study, contact information if there were any questions, and a link for parents to complete an online survey if they were interested in participating.

Forty-one families were solicited at the first childcare facility and 130 families were solicited at the second facility. These parents selected the link and were first exposed to the consent form. Participants could not proceed with the online survey unless they provided their consent. For the online survey parents answered questions regarding their children’s developmental milestones and also provided screenshots of their smartphone use. Parents also answered questions regarding their children’s mobile media use. The online survey took approximately 20–30 min to complete, and parents were paid $10 for completing the survey. All aspects of this study were approved by the appropriate institutional review board, and all parents provided consent for themselves and their children.

### 3.2. Participants

Having recruited from two childcare facilities, 63 participants completed the online survey. Based on a G*Power (v 3.1) analysis, with an effect size of 0.20, alpha probability of error 0.05 and power of 0.80, this exceeded the number of participants needed to detect significant differences (*n* = 59). The average age of parents was 34.62 (SD = 6.31) and the average age of their children was 3.74 (SD = 1.19). The majority of parents completing the online survey were mothers (79.4%), followed by 19.0% fathers, and 1.6% included a non-biological caregiver. Participants’ ethnicities were as follows: 74.6% white, 11.1% Hispanic, 6.3% black, 4.8% other, and 3.2% Asian, representing the demographics of the area in which they were recruited. There were no significant differences in demographics based on the two different facilities in which participants were recruited.

### 3.3. Measures

Means, standard deviations, and correlations are presented in Table 1.

#### 3.3.1. Children’s Developmental Milestones

Parents completed the ages and stages questionnaire (ASQ-3) [1]. This tool asks six questions on five different domains of children’s development: gross motor, fine motor, communication, problem solving, and personal–social. Each set of six questions considers the age of the child up to the month, which reflects the developmental milestone for that age [1,43]. For example, for an 18-month child regarding their gross motor skills, one of the questions is “Does your child walk well and seldom fall?” and for a 54-month child, a question regarding gross motor skills is “Without holding onto anything, does your child stand on one foot for at least five seconds without losing their balance and putting their foot down?” Options for all five domains are yes (10), sometimes (5), and not yet (0). This scale has displayed acceptable construct validity and reliability for children ages 0 to 5 [43]. Only the domains of motor and personal–social skills were included in the final study, as the other domains required open-ended responses that were not able to be captured to determine reliability. The reliability values (i.e., Cronbach’s alpha) across these three domains are 0.88 for gross motor skills, 0.89 for fine motor skills, and 0.79 for personal–social skills.

#### 3.3.2. Parent’s Mobile Media Use

Parents submitted screenshots of the last 24 h of their mobile media use. These screenshots included the applications that parents used and the number of minutes that parents used their device. It is important to note that all parents had a smartphone even though this was not an inclusion criterion for this study. To confirm the information from their screenshots, parents were also instructed to total the amount of time they were on their phone for the last 24 h and disclose that information as an open-ended question. There were no discrepancies between the screenshots and what parents entered into the open-ended box.

#### 3.3.3. Children’s Mobile Media Use

Parents reported the number of minutes they perceived their children to use a smartphone and/or tablet (cumulatively). Parents were also asked to submit a screenshot of their children’s most-used device to indicate how many minutes their children spent total on that device. If children did not have a device, but used their parents, then parents indicated, out of the total time indicated on their screenshots, how many of those minutes involved the child using this device.

#### 3.3.4. Parent–Child Co-Viewing

Using the information from the screenshots that parents provided of their own and their children’s device, parents indicated what percent of the time parents and children viewed the device together (out of 100%).

#### 3.3.5. Other Variables

In addition to children’s mobile media use, parents also indicated approximately how many minutes their children used television on an average day. Additionally, parents’ provided their age and their level of education from 0 (less than high school) to 14 (advanced graduate degree, such as M.D., Ph.D., etc.).

### 3.4. Data Analysis

Data for this study were first analyzed using correlational analyses to indicate the relationships across all study variables. Second multilevel regression analyses were conducted. The first step included the control variables, including parents’ age and education; the second step included children’s time watching television, using a laptop or tablet, using a smartphone, and parents’ total mobile media time; and the third step included percent time of children’s and parents’ time using mobile technology that was co-viewing. The dependent variables for these regression analyses were three of the five domains from the ASQ-3: fine motor skills, gross motor skills, and personal–social skills. Effect size was captured using *R*^2^ to identify the variability explained by the models. Prior to running analyses, Shapiro–Wilks and Kolmogorov–Smirnov tests were conducted to ensure that the data met the assumption for normality; all data did not deviate from normality (*p* > 0.05 for all variables).

## 4. Results

The goal of this study was to understand the relationship between children’s and parent’s mobile media use for children’s developmental milestones. First, correlations were conducted (see Table 1). Children’s own mobile media use was not correlated with any of the developmental milestones under investigation. However, parents’ mobile media use was positively associated with children’s gross motor (r = 0.36, *p* < 0.01) and children’s personal–social skills (r = 0.31, *p* < 0.05).

Next, regression analyses were conducted to test the hypotheses. Results of these analyses are presented in Table 2. Parent mobile media use was positively associated with children’s gross motor skills (B = 0.38, *p* < 0.01) and children’s personal–social skills (B = 0.36, *p* < 0.01), but not associated with children’s fine motor skills. Children’s television time was negatively associated with children’s gross motor skills (B = −0.30, *p* < 0.05), but not associated with children’s fine motor skills or personal–social skills. Children’s mobile technology use (tablet, laptop, or smartphone) was not associated with any measures of children’s developmental milestones. These results provide partial support for the first two hypotheses.

The third hypothesis sought to understand the role of parent–child co-viewing technology together for children’s milestones. This information is also presented in Table 2. Parents co-viewing technology with their children was positively associated with children’s fine motor skills (B = 0.32, *p* < 0.05). Parent–child co-viewing technology was not significantly associated with children’s gross or personal–social skills, although they were trending towards significance (*p* < 0.10), where co-viewing was negatively associated with children’s gross motor and personal–social skills. The effect sizes of these models were small, ranging from 0.09 to 0.19, meaning that 9–19% of the variance in children’s motor and personal–social skills was explained by these variables.

## 5. Discussion

The goal of this study was to better understand the relationship between parents’ and children’s individual and co-viewing use of technology for children’s developmental milestones. Research has illustrated that television use is generally bad for children’s development [6,18,44], but the findings of mobile technology are mixed [6], although trending towards a negative relationship. This study extends the research by examining different forms of technology use for children to help understand the similarities and differences between television and mobile media use for children’s development, while also adding parent–child co-viewing as a possible predictor of child development. Results of this study showed that there were no relationships between children’s mobile media use and their own development, but their television use was negatively associated with their gross motor skills. Additionally, parent–child co-viewing was positively associated with fine motor skills, trending towards negative associations with gross and personal–social skills. These results display the nuanced effects of technology for children’s development.

This study concluded that children’s mobile media use did not have any direct relationships with their developmental milestones as hypothesized. There are some explanations for this null finding. First, the time spent on mobile media is likely less important than how the media device is used. There are varying forms of mobile media content that children have access to, some being more educational than others. There is some evidence that applications that are more socially interactive are more beneficial for children’s development [45]. This finding should not take away from the fact that actual screen time does matter, but in contrast, content of screentime should be considered in future research [46]. Second, there are other possible control variables that could contribute to children’s developmental milestones beyond use of devices, such as the amount of time children play [47], parenting styles [48], and the frequency of parent–child interactions [49]. Future studies should consider these other variables to get a better sense of the contributions of media use for children’s development. Third, it is also possible that the consequences of mobile media use may be latent, not showing up until later in development. Longitudinal research is needed to best understand the relationships between technology use and children’s developmental milestones.

Another variable that could explain the null finding that was examined in this study was parent–child co-viewing. With parents having the opportunity to co-view with their children, there is space for the zone of proximal development to take place and scaffolding to occur [50]. Children learn at their own pace, but when they have a teacher, particularly a parent or caregiver, to steer them in the right direction, technology can be useful for learning [51]. Modeling certain tasks, such as when an application has an interactive touch screen feature, can display fine motor skills for the child to replicate [52]. When first learning a concept through an educational application, there are opportunities for questions and guided critical thinking to take place [53], particularly if these opportunities are scaffolded [50]. It is also important to consider supervising what children consume on mobile media to ensure safety and education [51,53]. Although co-viewing is valuable, it is not necessarily helpful for gross motor and personal–social skills since using mobile devices usually does not involve a lot of large body movements like running, jumping, balancing, or climbing [54]. Even though co-viewing of technology has a social aspect, it does not replicate social interaction with kids their age and social skills such as pretend play, turn-taking in conversation, or navigating peer conflict [46]. We did not measure the amount of or frequency of interaction between children and parents when co-viewing technology. Future studies are encouraged to examine the frequency and intensity of socializing and scaffolding that occurs when parents and children use a mobile device together.

Although there were null findings with mobile media, the use of television was more transparent based on the results of this study, which showed a negative relationship with gross motor skills. Watching television can be detrimental to gross motor skills because it is a passive activity, involving sitting or lying down for extended periods [44]. Movement-based play is essential for young kids as they learn sensorimotor feedback as they receive feedback from their environments and social contexts. Video games can also fit into this category of lacking real face-to-face social interaction and physical movement [55]. There is research evidence that children who spend more time on screen media have increasingly larger delays in motor development [55]. Early childhood is a critical window for these skills to emerge, making it important to prioritize movement-rich environments over screen-based ones. Gross motor skills develop through repetition and muscle use; every hour spent in front of a screen is an hour not spent playing outdoors, riding a bike, or engaging in active exploration, all of which are key to building core strength, coordination, and balance [56]. Each child has a critical window to learn these basic physical movements and if mobile media and television use is high, there may be delays in motor development in the future [56].

It is important to note that parents’ mobile media use was associated with children’s developmental milestones, which contradicts the literature. This unexpected finding could be explained by contextual factors. For example, parents may use their device as a way to support their child’s development, such as reading articles about child development, researching activities to support their child’s development or coordinating opportunities for children to engage with other children. Parents may also use their devices to engage in physical activities together, such as dancing to music from a YouTube video or talking to someone else through a video call, which could help support children’s development [57]. It is also possible that parents who use their device more may work from home or have flexible work schedules; these affordances of their job could provide parents more opportunities to engage with their children. Future studies should untangle the context in which parents use their device to better understand these results.

The results of this study also have theoretical implications. Vygotsky’s sociocultural theory of cognitive development emphasizes the crucial role that social interaction and cultural context have in children’s cognitive development [13]. According to Vygotsky’s sociocultural theory, children’s motor and social development emerges through guided interaction within their zone of proximal development (ZPD), where learning is guided by more knowledgeable individuals to complete a task with their assistance, often caregivers or teachers [50]. In the context of screen media use when regarding the ZPD, caregivers can heavily influence not only how often children engage with screens, but also the quality and context of that engagement [50]. Including parenting strategies such as setting boundaries, co-viewing, and selecting developmentally appropriate content, caregivers scaffold children’s understanding and regulate their experiences with media [58]. Supporting learning proximally and having interactive moments is crucial, as each child will model the examples that are displayed to them [6].

The results of this study can be used to provide recommendations for families regarding family mobile media use. First, it is important to set boundaries for screen time. Second, it would be beneficial to avoid ‘free range’ applications such as YouTube or other social media platforms, as they are not beneficial for preschoolers’ development [6]. Depending on the age of the child, it is recommended to have child-focused protections or guidelines embedded into the settings of applications that children use to ensure that they spend more time being active and social, especially with parents. It is most important for preschoolers to engage in play with their parents and caregivers, which is strongly supported by the literature [59]. Third, joint parent–child engagement (whether viewing media or not) is recommended to allow children to experience Vygotsky’s zone of proximal development and learn through guided practice and modeling [13]. Feedback through guided learning can aid children in feeling confident in their abilities and comfortable to ask questions about concepts they may have confusion or uncertainty about.

### Limitations and Conclusions

Although this study advances the literature on children’s mobile media use and development, it is not without its limitations. First, this study relied on parents’ perceptions of their children’s developmental milestones, which are prone to social desirability bias. Additionally, the sample was recruited from the same county in southern central United States, which limits generalizability. Also, although there was enough statistical power to analyze data in this study, larger samples are needed to better understand the relationships under investigation. These limitations may explain the small effect sizes of the statistical models in this study, which ranged from 9 to 19%. More sophisticated models with larger and more diverse samples could potentially strengthen these models. Also, the two ontogenetic periods (0–2 versus 3–5) are developmentally different; it may be beneficial to compare these two groups, which we did not have enough power to test. As mentioned, longitudinal studies that consider additional control variables would advance the literature on children’s mobile media use, and larger sample sizes could allow for in-group comparisons. This study also relied on cross-sectional data, which does not allow for cause-and-effect inferences. There were also some control variables that were not included in these models, such as children’s age and gender. Although developmental milestones should improve by age, focusing on this variable may provide more context for the findings in this study. Although there is little support for gender differences, the contribution of children’s technology use could vary by gender. Future studies should apply more advanced methodological designs to understand the relationships between children’s and parents’ mobile media use for children’s development.

Regardless of these limitations, this study provides an initial examination for how mobile media is similar and different regarding children’s development. Mobile media provides opportunities for parents and children to work and communicate together, and children can practice their fine motor movement. The negative consequences of television viewing are similar and different from mobile media use. Information from this study can inform future policies regarding children’s mobile media exposure and use, while informing the importance of parents’, teachers’, and others’ involvement with children’s mobile media use.

## Figures and Tables

**Table 1:** Means, standard deviations, and correlations across study variables.

Variables	Mean	SD	1	2	3	4	5	6	7
1. Gross motor skills	9.56	4.46	---	0.65 **	0.70 **	0.36 **	−0.07	0.15	0.11
2. Fine motor skills	7.34	3.66		---	0.70 **	0.15	0.09	0.20	0.15
3. Personal–social skills	8.24	3.67			---	0.31 *	0.04	0.22	0.07
4. Parent mobile media minutes	170.57	153.82				---	−0.09	−0.13	−0.03
5. Child television minutes	32.29	46.36					---	−0.04	0.12
6. Child tablet/laptop minutes	12.25	24.89						---	0.00
7. Child smartphone minutes	4.86	12.29							---

** *p* < 0.01; * *p* < 0.05.

**Table 2:** Regression predicting children’s developmental milestones.

Variable	Gross Motor Skills	Fine Motor Skills	Personal–Social Skills
Intercept	8.10 (3.38) **	5.33 (2.90) **	5.68 (2.80) **
Parent age	−0.04 (0.10)	0.02 (0.08)	0.02 (0.08)
Parent education	0.15 (0.34)	0.14 (0.31)	0.09 (0.22)
Parent mobile media time	0.38 (0.00) **	0.20 (0.01)	0.36 (0.00) **
Child television time	−0.30 (0.01) *	0.11 (0.01)	0.08 (0.01)
Child tablet/laptop time	0.19 (0.02)	0.18 (0.02)	0.23 (0.02)
Child smartphone time	0.13 (0.05)	0.13 (0.04)	0.06 (0.04)
%time parent–child co-view	−0.20 (0.07)	0.32 (0.09) *	−0.19 (0.05)
*R* ^2^	0.19	0.09	0.17

Note: Data is presented as standardized beta coefficients with standard errors in parentheses. ** *p* < 0.01; * *p* < 0.05.

## Data Availability

The raw data supporting the conclusions of this article will be made available by the authors on request.

## Abbreviations

The following abbreviations are used in this manuscript:

ASQ	Ages and Stages Questionnaire
